# The molecular structure of the glycoside hydrolase domain of Cwp19 from *Clostridium difficile*

**DOI:** 10.1111/febs.14310

**Published:** 2017-11-17

**Authors:** William J. Bradshaw, Jonathan M. Kirby, April K. Roberts, Clifford C. Shone, K. Ravi Acharya

**Affiliations:** ^1^ Department of Biology and Biochemistry University of Bath UK; ^2^ Public Health England Salisbury UK

**Keywords:** bacterial adhesion, cell wall, *Clostridium difficile*, colitis, crystal structure

## Abstract

*Clostridium difficile* is a burden to healthcare systems around the world, causing tens of thousands of deaths annually. The S‐layer of the bacterium, a layer of protein found of the surface of cells, has received a significant amount of attention over the past two decades as a potential target to combat the growing threat presented by *C. difficile* infections. The S‐layer contains a wide range of proteins, each of which possesses three cell wall‐binding domains, while many also possess a “functional” region. Here, we present the high resolution structure of the functional region of one such protein, Cwp19 along with preliminary functional characterisation of the predicted glycoside hydrolase. Cwp19 has a TIM barrel fold and appears to possess a high degree of substrate selectivity. The protein also exhibits peptidoglycan hydrolase activity, an order of magnitude slower than that of lysozyme and is the first member of glycoside hydrolase‐like family 10 to be characterised. This research goes some way to understanding the role of Cwp19 in the S‐layer of *C. difficile*.

**Database:**

Structural data are available in the PDB under the accession numbers 5OQ2 and 5OQ3.

**Abbreviations**
CWBD: cell wall‐binding domain
Cwp: cell wall protein
LMW SLP: low‐molecular weight S‐layer protein

## Introduction

The Gram‐positive “superbug” *Clostridium difficile* has received significant media attention in recent decades as the primary causative agent of antibiotic‐associated diarrhoea. More severe infections can lead to pseudomembranous colitis and toxic megacolon 1. Increasing levels of antibiotic resistance mean that the threat from *C. difficile* is also increasing 2, 3. The bacterium presents a significant burden to healthcare systems, causing tens of thousands of deaths globally each year 4. This demonstrates that a greater understanding of the bacterium is required for the development of novel strategies to combat *C. difficile* infections.

The bacterium presents a layer of protein on the surface of the cell known as an S‐layer 5, 6. S‐layers have been shown to possess a range of important roles including, but not limited to, cell shape determination, molecular sieving, host cell adhesion and/or invasion, immune system evasion and protection from competing microorganisms 7. The S‐layer of *C. difficile* is primarily formed of the high‐ and low‐molecular weight S‐layer proteins (HMW SLP and LMW SLP, respectively), which are derived from the cleavage of the S‐layer precursor protein, SlpA 8, 9. HMW SLP is responsible for binding to the cell wall and possesses three cell wall‐binding domains (pfam 04122, CWB2). There are 28 SlpA paralogues in the *C. difficile* genome, each of which possesses three CWB2 domains, and many also possess “functional” regions 5, 10, 11. Understanding the structure and function of the range of proteins within the S‐layer of *C. difficile* is of major importance if the S‐layer is to be exploited as a drug target.

One of the proteins contained within the S‐layer of *C. difficile*, Cwp19, has been determined by Pfam and BLAST to contain a glycoside hydrolase‐like 10 (GHL10) domain with a high degree of certainty (E = 10^−93^). Pfam also gives a potential classification within the same region of a family 27 glycoside hydrolase (GH27), while a BLAST search also suggests a GH36. Both of these classifications, however, have much lower degrees of certainty than GHL10 12, 13, 14. GHL1‐GHL15 were identified in 2011 as families of proteins that are likely to exhibit glycoside hydrolase activity and possess a triosephosphate isomerase (TIM) barrel fold 15, a common eight‐stranded β‐barrel exhibited by many glycoside hydrolases and a wide range of other proteins 16. GHL1 has since been reclassified as GH129 17, while the remaining GHL families are yet to be characterised.

Pfam reports that over 1000 protein sequences have been classified as containing GHL10 domains, of these, around 1% have been identified in fungi and animals, while the remaining 99% are spread across a wide range of bacterial phyla.

The gene coding for Cwp19 is located in the anionic polymer locus (AP locus) which is likely to be involved in synthesis of PSII, the polysaccharide that mediates binding of CWB2 domains to the cell wall. The AP locus is itself immediately downstream of the *slpA* locus, which contains the first twelve *cwp* genes and six others with apparent roles relating to the S‐layer or cell wall 8, 9, 18, 19. It is therefore possible that Cwp19 will be involved in processing surface exposed polysaccharides such as peptidoglycan or PSII.

*cwp19* has been shown to be present with more than 95% amino acid sequence identity in a wide variety of *C. difficile* strains 18, 20. Although expression is yet to be thoroughly analysed, Cwp19 is known to be present in the S‐layer under at least some conditions as it copurified with the cysteine protease Cwp84 in a pull‐down assay using probes based on E‐64, a cysteine protease inhibitor 21. A recent study on seven Brazilian *C. difficile* strains found that Cwp19 was the most abundant component in S‐layer extracts from three strains and was second only to Cwp2 in two strains and SlpA in one 22.

Here, we present the high resolution crystal structure of the functional region of Cwp19, referred to as Cwp19‐fr, and a selenomethionine‐derived structure used for phasing along with preliminary functional analysis towards elucidation of the role of Cwp19 in the S‐layer of *C. difficile*.

## Results

### The structure of Cwp19‐fr

**Figure 1:**
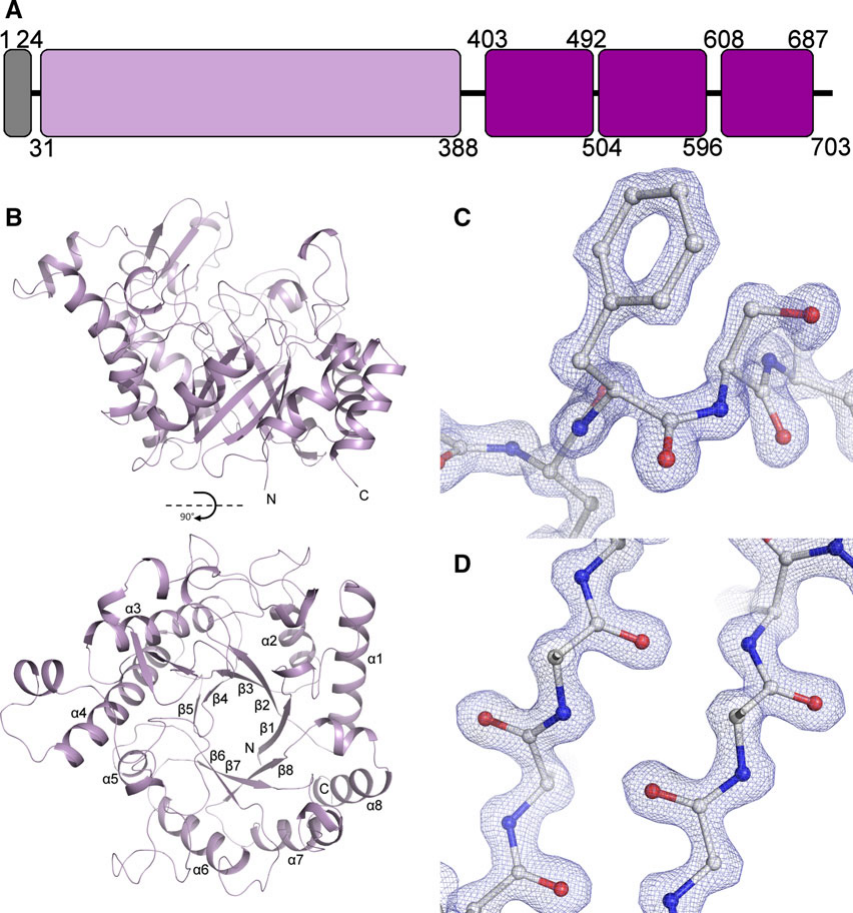
The Structure of Cwp19‐fr. (A) Domain representation, the signal peptide is coloured grey, the GHL10 domain is lilac and the cell wall‐binding domains are purple. The present construct codes for residues 27‐401, while residues 28‐388 are visible in the structures. (B) Ribbon diagram of the overall fold. Cwp19‐fr assumes a TIM barrel fold, forming an eight‐stranded β‐barrel surrounded by eight α‐helices. The active site is formed over the centre of the barrel near the C‐termini of β‐strands. The two images are related by a 90° rotation on the *x*‐axis. (C) Example electron density, 2F_O_‐F_C_, 2.5σ. The cis‐peptide formed by Phe367 and Ser368 is shown. (D) Sections of β‐strands 5 and 6.

The structure of Cwp19‐fr has been determined by selenium single‐wavelength anomalous diffraction (Se‐SAD) and to a high resolution with native data using a construct coding for residues 27‐401, although electron density is only visible for residues 28‐388 across the two structures presented here. This construct coded for the predicted glycoside hydrolase‐like family 10 domain (Fig. 1A). Crystallographic and refinement statistics are summarised in Table 1. This construct does not contain the signal peptide, which is predicted to be cleaved between residues 24 and 25 23, or the three C‐terminal cell wall‐binding domains, the first of which is predicted to start at residue 402 14.

**Table 1:** Crystallographic and Refinement Statistics. Inner shell statistics are given in square brackets, overall statistics are unbracketed and outer shell statistics are given in round brackets

	Se‐SAD	High resolution native
Crystallographic statistics		
Space group	P2_1_	P2_1_2_1_2_1_
Cell dimensions (Å, °)	55.3, 60.4, 105.1 90, 94.2, 90	62.0, 65.6, 104.0 90, 90, 90
Resolution (Å)	[55.13–8.91] (2.38–2.30)	[65.64–7.39] (1.37–1.35)
R_merge_	[0.142] 0.255 (0.591)	[0.067] 0.118 (0.674)
R_meas_	[0.145] 0.260 (0.603)	[0.069] 0.123 (0.704)
R_pim_	[0.028] 0.050 (0.116)	[0.019] 0.033 (0.200)
CC_1/2_	[0.998] 0.999 (0.980)	[0.998] 0.999 (0.982)
Mean <I/σI>	[68.5] 28.2 (9.9)	[40.4] 15.4 (3.7)
Completeness (%)	[99.7] 100.0 (100.0)	[99.8] 99.5 (100.0)
Total number of reflections	[26 570] 1 631 844 (162 160)	[14 762] 2 376 913 (107 557)
Total number of unique reflections	[573] 30 986 (3026)	[679] 93 318 (4543)
Multiplicity	[46.4] 52.7 (53.6)	[21.7] 25.5 (23.7)
Anomalous completeness (%)	[99.8] 100.0 (100.0)	[100.0] 99.4 (100.0)
Anomalous multiplicity	[26.0] 26.6 (26.9)	[13.1] 13.3 (12.0)
CC_anom_	[0.620] 0.448 (0.203)	[−0.484] −0.307 (−0.195)
Anisotropic delta‐B	9.07	11.58
Anisotropic CC_1/2_ = 0.3 (Å)	1.95, 1.78, 1.53	1.04, 1.32, 0.99
Refinement statistics		
R_work_/R_free_	0.192/0.254	0.149/0.174
RMSDs		
Bond Lengths (Å)	0.009	0.013
Bond Angles (°)	1.312	1.535
Ramachandran Statistics (%)		
Favoured	94.8	97.2
Allowed	4.5	2.8
Outliers	0.7	0
Average B‐factors (Å^2^)		
Protein	28.8	15.0
Ligand	46.6	25.8
Water	21.2	28.1
Number of atoms		
Protein	5788	2940
Ligand	14	12
Water	136	385
PDB Code	5OQ2	5OQ3

The Se‐SAD structure of Cwp19‐fr has been determined to a resolution of 2.3 Å and contains two protein chains in the asymmetric unit with two phosphate ions, a PEG molecule and 136 water molecules, while the high resolution native structure has been determined to 1.35 Å with one protein chain in the asymmetric unit, two PEG molecules, a chloride ion and 385 water molecules. The two Se‐SAD Cwp19‐fr chains superpose on the high resolution structure with RMSDs of 0.27 Å (2527 atoms) and 0.28 Å (2435 atoms), while they superpose on each other with an RMSD of 0.28 Å (2408 atoms).

As predicted, Cwp19‐fr assumes a typical TIM barrel fold, forming an eight‐stranded parallel β‐barrel surrounded by eight α‐helices (Fig. 1). This structure is formed by a repeating βα motif. The TIM barrel is formed by residues 33‐388, it is assumed that residues 389 to approximately 401 form a disordered loop linking the TIM barrel to the first CWB2 domain. Loops following α‐helices and preceding β‐strands (αβ loops) on one side of the barrel are considerably shorter than those following strands and preceding α‐helices (βα loops) on the other. Longer βα loops than αβ loops is a common feature of TIM barrels. αβ loops frequently have the purely structural role of barrel formation, while βα loops show a significant amount of variation and form any functional sites on one side of the barrel 16.

### Identification of the active site

**Figure 2:**
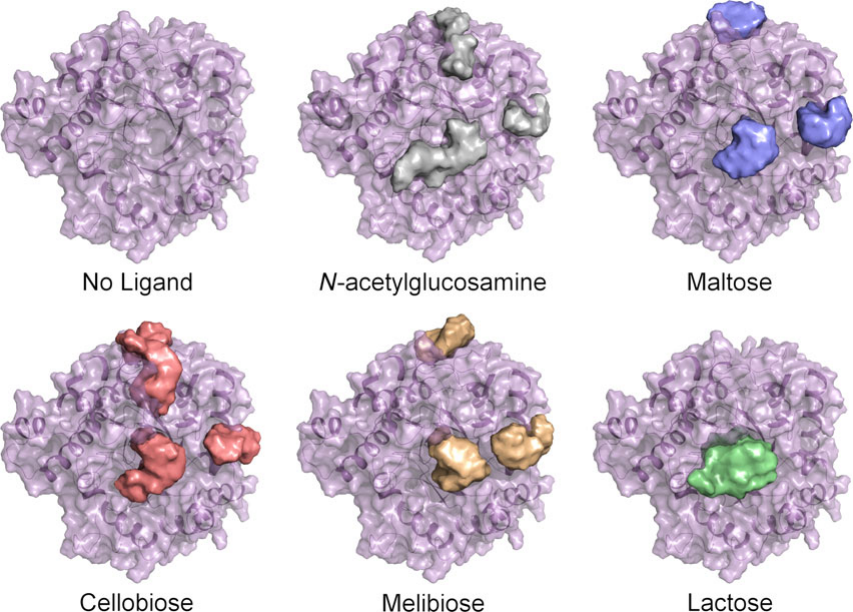
Docking results. Cwp19‐fr is shown with the docking results for one monosaccharide and four disaccharides. Each image shows a surface representation of approximately 250 docking results for the respective carbohydrate. This produces a shape within which a sugar is predicted to bind, from this the central active site can be identified and potentially also regions to which distal portions of the substrate are able to bind. These regions are shown above and to the right of the central active site region.

Docking of simple carbohydrates to the high resolution structure of Cwp19‐fr using SwissDock 24 gave around 1250 potential modes of substrate binding. The majority of the docked ligands sat roughly centrally over the barrel (Fig. 2). Although the intention of this exercise was not to determine exactly how any substrate binds, it does give a strong indication that this is the active site. This was further confirmed with a structural alignment using the DALI server 25 against Cwp19‐fr, which identified hundreds of structures with significant Z‐scores (Z > 2.0). PgaB (Carbohydrate esterase family 4), a subunit of a poly‐β‐1,6‐N‐acetylglucosamine deacetylase from *E. coli* 26 was the closest match (Z = 23.8–26.2), followed by *Bifidobacterium bifidum* β‐galactosidase (GH42, Z = 22.7) 27 and *Solanum lycopersicum* β‐mannanase 4a (GH5, Z = 22.0) 28. These structures showed a conserved active site in the same location as that identified by the docking. Interestingly, the putative active site in the high resolution structure shows a small amount of strong unidentified density, a formate ion fits the density well but no formate was known to be included in the crystallisation conditions, so the density was left uninterpreted.

### Peptidoglycan hydrolase assay

**Figure 3:**
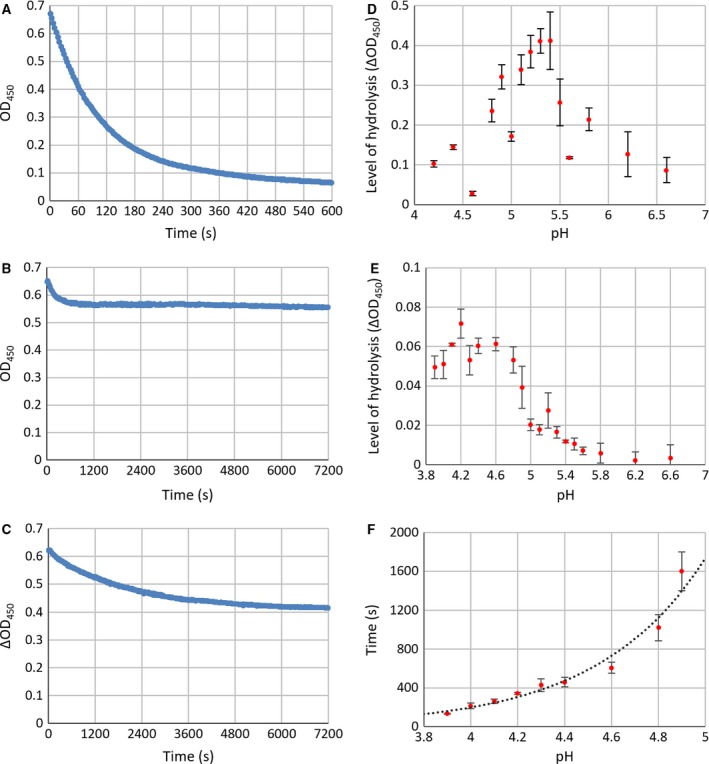
Peptidoglycan hydrolase activity assay results. (A) Lysozyme control. The rate of hydrolysis by lysozyme causes almost all cells to be lysed within 10 min. (B) Example lysis by Cwp19 at pH 4.4. The rate of lysis is initially fast, but the reaction stops after a short length of time. (C) Example lysis by Cwp19 at pH 5.0. The initial rate is slower than at pH 4.4, but the reaction continues for much longer allowing more Cwp19 to be hydrolysed. (D) ΔOD_450_ relative to starting OD_450_ against pH over 2 h. The greatest change in OD and therefore the greatest degree of lysis over 2 h was observed between pH 5.2 and 5.4. (E) ΔOD_450_ relative to starting OD_450_ against pH over 2 min. A considerably faster initial rate was seen at more acidic pHs. This could not be investigated beyond pH 3.9 as the cells appeared to spontaneously lyse. (F) Time before the reaction stopped against pH. As the rate was slower at more basic pHs, it became more difficult to determine a point of cessation, demonstrated by the larger error bars, so only pHs between 3.9 and 4.9 have been included (*n* = 3, all error bars are SEM).

It has previously been suggested that Cwp19 is capable of breaking down peptidoglycan (Peltier *et al*. unpublished work). This was used as a starting point for the determination of an optimum pH for Cwp19‐fr at which further activity assays could be performed. The lysis of *Micrococcus luteus* cells, measured as the change in OD_450_ of a cell suspension due to peptidoglycan breakdown was used to assess peptidoglycan hydrolase activity. Due to variations in the initial OD, lysis was calculated as a proportion of the initial OD. Hydrolysis caused by the action of lysozyme at pH 6.2 was used as a control. Lysozyme showed a rapid breakdown with a linear rate over the first 30 s with an average of 7.4 × 10^−3^ ± 1.1 × 10^−4^ s^−1^ (SEM) (Fig. 3A). Peptidoglycan hydrolysis was measured in the presence of Cwp19‐fr at pHs between 3.9 and 6.6 over 2 h. The decrease in OD over the first 2 min was observed to be largely linear, so this was used to calculate initial rates. A faster initial rate was observed at the more acidic pHs, but the reaction stopped after a short length of time (Fig. 3B), while it continued for longer at less acidic pHs (Fig. 3C). To determine whether the halting of the reaction was due to a lack of stability of Cwp19‐fr in a more acidic environment, the protein was incubated at pH 3.9 for 30 min before the reaction was started by the addition of cells. The reaction proceeded as normal, but continued for the entire 15 min of the assay. The fastest initial rate of 5.8 × 10^−4^ ± 1.1 × 10^−4^ s^−1^ was measured at pH 4.2, approximately 13 times slower than that of lysozyme. Monitoring of the reaction over 2 h produced a clear bell‐shaped curve centred around pH 5.2–5.4 (Fig. 3D). The initial rate at pH 5.3 was measured to be 1.2 × 10^−4^ ± 4.0 × 10^−5^ s^−1^, 66 times slower than lysozyme. The initial rate of reaction appeared to show a plateau around approximately pH 4.3 (Fig. 3E), but due to apparent spontaneous rapid lysis of the cells at pHs below 3.9, this plateau could not be confirmed. While the time before the reaction stopped was seen to increase exponentially with pH (Fig. 3F).

### Benedict's assay

**Figure 4:**
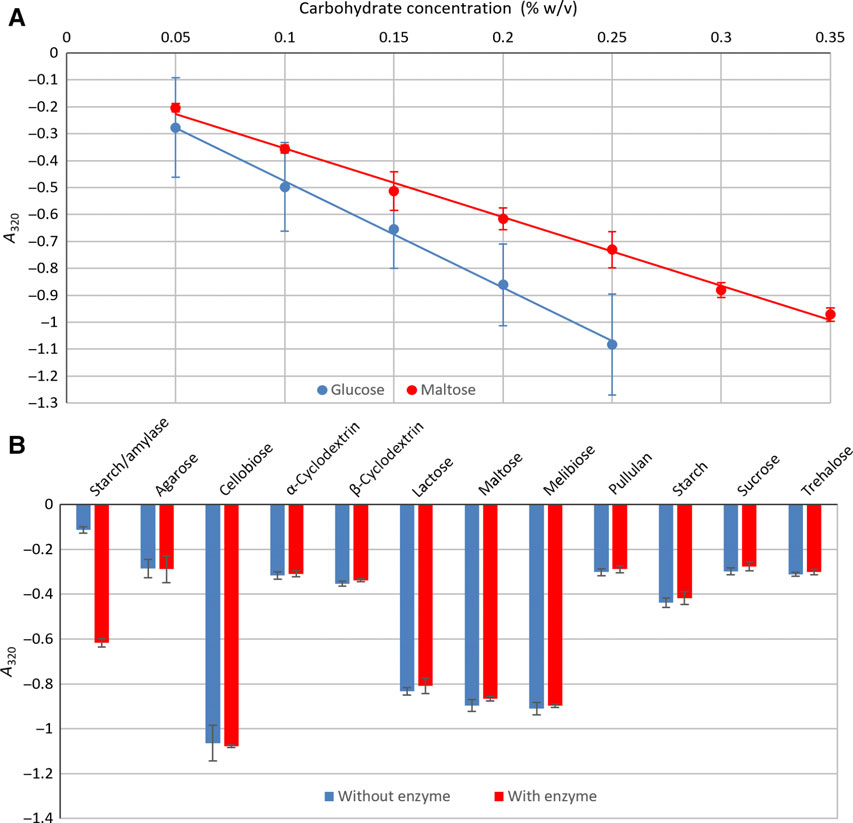
Benedict's assay. (A) Conformation that Benedict's test can be used to differentiate between a monosaccharide and a disaccharide. Glucose clearly results in a greater decrease in A_320_ than an equal concentration of maltose. (B) Detection of carbohydrate breakdown. A significant difference was observed in the starch/amylase control (Student's *T*‐test, *P* < 0.001), but no difference (*P* > 0.05) was seen for Cwp19‐fr tested against any carbohydrate (*n* = 3, all error bars are SEM).

To demonstrate that Benedict's test can be used to distinguish between solutions of a disaccharide and a monosaccharide at equal (weight/volume) concentrations and to determine sensible concentrations at which to perform the assay, Benedict's test was performed with a range of concentrations of glucose and maltose. Samples were zeroed against equally diluted Benedict's with no carbohydrate. The presence of carbohydrate resulted in a decrease in the concentration of copper (II) and therefore, “blueness”, which was measured as a decrease in A_320_. A difference was also observed at approximately 735 nm, however, this was not as significant as the change at 320 nm. This showed a well correlated linear relationship between carbohydrate concentration and A_320_ and a clearly observable difference between the two carbohydrates (Fig. 4A). The observed linearity stopped at an A_320_ of approximately ‐1, which was likely to have been a result of exhaustion of copper (II). This absorbance equated to a glucose concentration of around 0.25%, so this concentration was selected for the assay. The ability of Cwp19‐fr to break down 11 different carbohydrates was assessed (Table 2). Starch was broken down with amylase as a positive control, this showed a significant difference between the sample with amylase and without (Student's *T*‐test, *P* < 0.001). None of the 11 carbohydrates showed significant changes in absorbance in the presence of Cwp19‐fr (*P* > 0.05, Fig. 4B).

**Table 2:** Carbohydrates used for Benedict's assay. All monosaccharides with unexposed reducing ends are in pyranose forms except fructose in sucrose, which is in the furanose form

Carbohydrate	Monosaccharides and bonds present
Agarose	[‐D‐galactose‐β‐1,4‐(3,6‐anhydro‐L‐galactose)‐α‐1,3‐]_n_
Cellobiose	D‐glucose‐β‐1,4‐D‐glucose
α‐cyclodextrin	Cyclo‐[‐D‐glucose‐α‐1,4‐]_6_
β‐cyclodextrin	Cyclo‐[‐D‐glucose‐α‐1,4‐]_7_
Lactose	D‐galactose‐β‐1,4‐D‐glucose
Maltose	D‐glucose‐α‐1,4‐D‐glucose
Melibiose	D‐galactose‐α‐1,6‐D‐glucose
Pullulan	[‐D‐glucose‐α‐1,4‐D‐glucose‐α‐1,4‐D‐glucose‐α‐1,6‐]_n_
Starch	[‐D‐glucose‐α‐1,4‐]_n_…D‐glucose‐α‐1,6‐D‐glucose…
Sucrose	D‐glucose‐α‐ β‐1,2‐ D‐fructose
Trehalose	D‐glucose‐α‐ α‐1,1‐ D‐glucose

## Discussion

Polysaccharides can essentially be divided into two groups based on their functions: energy storage and cellular structure. Glycoconjugates, on the other hand, usually have higher order functions such as cell to cell interactions and modulation of activity. The large amount of isomerism exhibited by monosaccharides, coupled with a broad range of potential linkages, necessitates a great deal of diversity and specificity among enzymes that process carbohydrates including glycoside hydrolases, which hydrolyse said linkages 29.

It has been noted that the 30 GHs identified in *Mycobacterium tuberculosis* can be categorised into four broad functional groups: metabolism of α‐glucans produced by the bacterium, peptidoglycan maintenance, hydrolysis of β‐glucans (primarily those consumed by the host) and α‐demannosylation of proteins produced by the bacterium as a method of functional modulation 30. It stands to reason that the majority of GHs in other bacterial species are likely to fit into similar categories. As *cwp19* is located in the AP locus, there is a significant possibility that it will be involved in the metabolism of surface exposed polysaccharides, such as PSII or peptidoglycan.

This work has resulted in the determination of the high resolution structure of the functional region of Cwp19, which possess a TIM barrel fold with similarities to a wide range of other glycoside hydrolases. The diverse functions of glycoside hydrolases make it difficult to predict a function based upon the structure.

### Active site

**Figure 5:**
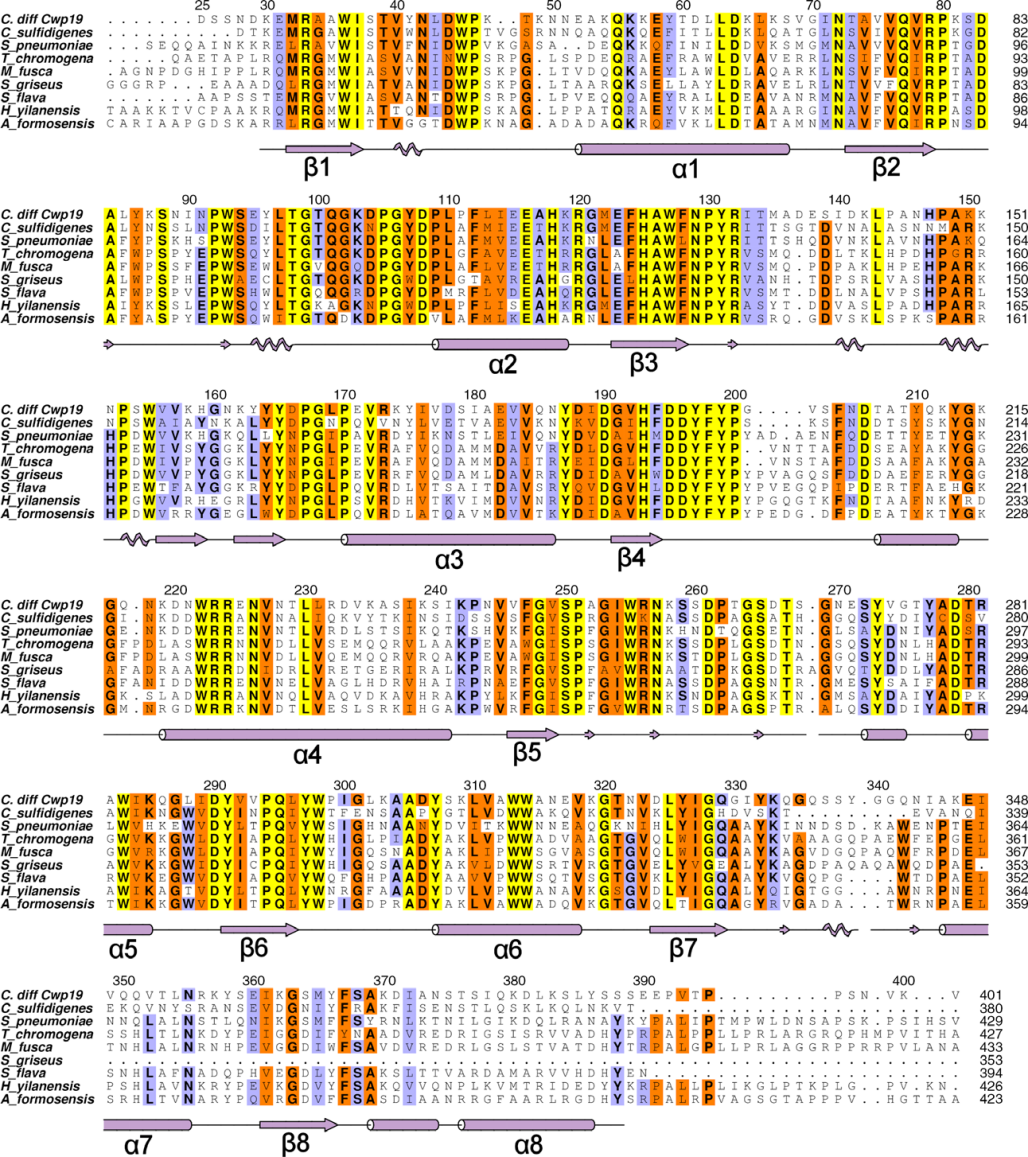
Multiple sequence alignment of *C. difficile* Cwp19_25‐401_ to GHL10 proteins identified by BLAST. Residues conserved across all sequences are highlighted in yellow, well conserved residues in orange, moderately conserved residues in blue. Cwp19 residue numbering is given above the alignment and secondary structure is given below. The eight β‐strands and α‐helices that form the TIM barrel are indicated, other α‐helices and β‐strands are not labelled, 3_10_ helices and β‐bridges are also indicated with zig‐zag patterns and small arrows respectively. Species and NCBI references are as follows: *Clostridium sulfidigenes*, WP_035134795.1; *Streptococcus pneumoniae*, COD97312.1; *Thermomonospora chromogena*, SDQ39562.1; *Microtetraspora fusca*, WP_066947673.1; *Streptomyces griseus*, WP_030738522.1; *Saccharopolyspora flava*, SFT07258.1; *Herbidospora yilanensis*, WP_062357460.1; *Actinomadura formosensis*, WP_067802682.1.

Probable active site residues have been identified using three methods, firstly, through docking experiments, which showed that the active site is likely to be positioned centrally over the barrel (Fig. 2). Secondly, through comparison to the closest structural homologues identified by DALI, whose active sites are also positioned over the centre of the barrel, confirming the location identified by docking, and finally through alignment to other proteins classified as GHL10 (Fig. 5) and by comparison to the GHL10 HMM logo available on the Pfam website (pfam.xfam.org).

Glycoside hydrolases can be classified based on whether they invert or retain (through two inversions) the stereochemistry of the substrate during catalysis. Both mechanisms involve a catalytic dyad, which is usually two acidic residues. In an inverting glycoside hydrolase, one acts as an acid, the other as a base, while in a retaining glycoside hydrolase, one acts as an acid in the first step and as a base in the second, while the other acts as a nucleophile, stabilising an intermediate 31, 32. Variations on the residues involved have been identified however, notably with a histidine residue acting as either an acid or a base in certain members of GH3 and GH117 29.

All three top DALI results identified Asp 196 as being an important residue. The equivalent in *E. coli* PgaB, Asp466, was suggested to be responsible for stabilisation of the catalytic oxazolinium intermediate 26 by comparison to the structures of acidic mammalian chitinase (GH18) 33 and dispersinB (GH20) 34. While Glu161 in β‐galactosidase was shown to be important to catalysis through mutagenesis and activity assays 27 and Glu204 from β‐mannanase 4a was identified as part of the catalytic dyad 28.

The other residue identified as part of the catalytic dyad in β‐mannanase 4a was Glu318, which is conserved in PgaB as Glu607 and *B. bifidum* β‐galactosidase as Glu320, which was similarly shown to be important through mutagenesis and activity assays. This residue is found at the C‐terminus of β7. In Cwp19‐fr, this strand is tilted away from the centre of the barrel and the residue is replaced with Gly328, which is conserved in GHL10. This results in a significantly different shape in this portion of the active site pocket of Cwp19‐fr. As this residue cannot be part of the catalytic dyad in Cwp19‐fr, it is likely that the substrate will be orientated somewhat differently in the active site of Cwp19‐fr, interacting with a different catalytic residue. Aside from the largely buried Asp195, no other acidic residues seem to be near enough to the active site or orientated in a way that they are likely to be catalytic. Considering the observed Asp‐His dyad in GH3 and GH117, this opens the possibility that the other catalytic residue may be basic. If this is the case, it appears that the only residue that could fit this role is Arg132, which is conserved in GHL10 (Fig. 5). In PgaB, the residues that form the dyad are 9.4 Å apart, while in Cwp19, Asp196 and Arg132 are 9.2 Å apart. Clearly, however, this level of conjecture requires further work to determine the role of various residues within the active site.

Tyr645, which is found at the C‐terminus of β8, was also identified in PgaB as being important to carbohydrate binding 26. β‐mannanase 4a was noted as possessing a cis‐peptide bond between the equivalent residue, Trp360, and Glu361 that was deduced to be important for the formation of the S_1_ pocket 28. This aromatic residue followed by a cis‐peptide is also seen in the other two DALI hits and Cwp19‐fr as well: between Phe367 and Ser368 (Fig. 1C). This cis‐peptide was also observed in the structures of *Triticum aestivum* xylanase (GH18) 35 and *Canavalia ensiformis* chitinase (GH18), and was noted as a “common characteristic of chitin‐binding proteins of family 18” that is likely to play a role in substrate binding 36. It is therefore possible that this cis‐peptide is also involved in the formation of the S_1_ pocket in Cwp19‐fr.

Another residue determined to be important in PgaB was Tyr432 which is found within the long β3‐α3 loop. Cwp19‐fr contains a similar extended loop, however it assumes a very different conformation. The position assumed by the side chain of Tyr432 in PgaB is, however, approximately replicated by Tyr197 in Cwp19‐fr, shortly following β4. This is adjacent to Asp196. The region surrounding these two residues shows a significant level of conservation (Fig. 5). Remaining portions of the binding site identified in PgaB are formed by loops β1‐α1 and β2‐α2, both of which assume different conformations in Cwp19‐fr.

The mutagenesis and activity assays on β‐galactosidase also identified Asn160, Tyr289 and His371 as important active site residues 27. Asn160 is conserved in GHL10 as Asp195 in Cwp19‐fr, although as noted for PgaB, the side chain of Asp195 is largely buried. Tyr289 is conserved in GHL10/Cwp19‐fr as Tyr297 at the C‐terminus of β6. His371 is near the centre of the β8‐α8 loop, which in Cwp19‐fr is replaced by a short nonconserved α‐helix and has no equivalent position.

**Figure 6:**
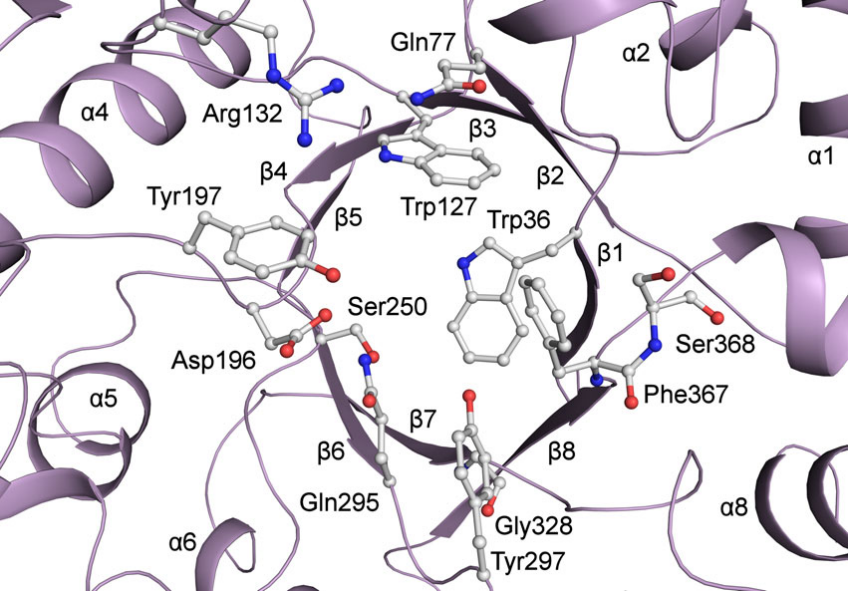
Cwp19‐fr active site. Residues identified based upon similarities to DALI hits and other conserved residues that form part of the putative active site pocket are shown. Notably, Asp196 is likely to either be involved in intermediate stabilisation or be part of the catalytic dyad, Gly328 replaces a catalytic glutamate, and the cis‐peptide bond formed by Phe367 and Ser368 may be involved in formation of the S_1_ pocket.

As well as the residues identified through inspection of DALI results, (Asp196, Tyr197, Tyr297, Gly328, Phe367 and Ser368), the alignment to other GHL10 proteins also allows the identification of Trp36, Gln77, Trp127, Arg132, Ser250 and Gln295 as conserved residues that are likely to be important to the formation of the active site and therefore substrate binding and/or catalysis (Fig. 6).

### Other sites highlighted based on docking study

As well as docking a large number of molecules to the putative active site, SwissDock also docked a significant number of molecules to two more peripheral regions. One of these regions is formed by loops β1‐α1 and β8‐α8, while the other is formed by loops β2‐α2 and β3‐α3. These loops show significant levels of variation in GHL10 proteins and are not conserved in the closest DALI hits. The HMM logo also shows possibilities of inserts in approximately these locations. It therefore stands to reason that these regions may be responsible for substrate specificity, allowing GHL10s to cleave a range of substrates. The cis‐peptide between Phe367 and Ser368, which is potentially involved in forming the S_1_ pocket, forms part of the connection between the active site and the β1‐α1 β8‐α8 groove. It is therefore possible that the portion of the substrate before the scissile glycosidic bond sits in the β1‐α1 β8‐α8 groove while the portion after the scissile bond sits in the β2‐α2 β3‐α3 groove.

### Activity measurements

Cwp19‐fr is able to cleave peptidoglycan at pHs between 3.9 and 6.6, with a maximum amount of product over 2 h formed at approximately pH 5.2–5.4 (Fig. 3D). A faster rate was initially observed at more acidic pHs, but it was not sustained (Fig. 3E). This effect clearly followed an exponential pattern strongly linking the time of onset of the arrest in activity to pH (Fig. 3F). The precise reason for this remains unclear. Interestingly, the slight decrease in OD_450_ that was observed at all pHs in the controls was not observed after the reaction had stopped.

Ultimately, the patterns observed here are complex and not enough information is available to fully explain them or to definitively determine an optimum pH. However, the fact that Cwp19‐fr is able to cause lysis of the cells appears to be clear. This is very likely to be due to the hydrolysis of peptidoglycan by Cwp19‐fr. However, it is not clear which of the two glycosidic bonds in peptidoglycan that Cwp19‐fr is capable of breaking down – N‐acetylglucosamine‐β‐(1,4)‐N‐acetylmuramic acid or N‐acetylmuramic acid‐β‐(1,4)‐N‐acetylglucosamine. As a significant amount of cells were lysed at pH 5.3, this pH was used for further assays.

It should be noted that even the fastest initial rate observed for Cwp19‐fr at pH 4.2 was approximately 13 times slower than that of lysozyme at pH 6.2, while at pH 5.3 the rate was approximately 66 times slower. This indicates that Cwp19 is unlikely to have a primary role of simply breaking down Gram‐positive peptidoglycan. *C. difficile* has been shown to possess an unusual form of peptidoglycan 37, so it is possible that the protein may act upon the bacterium's own peptidoglycan in a variety of possible roles.

The ability of Cwp19‐fr to break down a range of other carbohydrate substrates was also considered. Due to the observed slow breakdown of peptidoglycan, this assay was run for four hours. A decrease in A_320_ relative to the control samples indicated an increase in reducing ability of the sample, which was interpreted as an indicator of the ability of Cwp19‐fr to hydrolyse at least one type of glycosidic bond in the sample. A range of monosaccharide residues and glycosidic linkages were tested but no statistically significant results were observed. This suggests either that Cwp19 may act on a substrate or substrates not tested in this study, may only be capable of breaking down very specific substrates or that the rate of reaction for the substrates tested was too slow for a reaction to be observed. The presence of *cwp19* in the AP locus, which has been implicated in the formation of PSII, indicates a potential role for Cwp19 in the cleavage of a PSII precursor 18, 19.

### PXXP motif

Glycoside hydrolase‐like family 10 proteins possess a well conserved PXXP motif immediately preceding α3 – PGLP^170^ in Cwp19. Notably, SH3 domains, two of which are found in Cwp14 5, 10, 11, bind PXXP motifs 38, 39. It is therefore, possible that there may be an interaction between Cwp19 and Cwp14. The structure of Cwp19‐fr, however, reveals that this motif is largely occluded by the beginning of α4 and the loop preceding it, particularly a short α‐helix contained within the loop. A portion of the loop does possess slightly elevated B‐factors, but it is unlikely that it will be flexible enough to facilitate binding of Cwp14. The loop is, however, poorly conserved, so it is possible that the PXXP motif in other GHL10 proteins may bind to SH3 domains.

### Conclusions

The structure of the glycoside hydrolase domain of *C. difficile* S‐layer‐associated protein Cwp19 consisting of residues 28‐388 has been determined to a high resolution. This is the sixth structure of the functional region of a protein from the S‐layer of *C. difficile* to be determined after LMW SLP 40, Cwp84 41, 42, Cwp6 and Cwp8 43 and Cwp2 44. We have identified a number of potential residues that are likely to be important active site residues and have partially characterised the activity of Cwp19‐fr. This work adds to the growing picture of how this complex S‐layer works. Cwp19 is classified as belonging to glycoside hydrolase‐like family 10 based on sequence similarity. GH activity has now been confirmed, however precise substrates are yet to be determined. Further characterisation is needed before GHL10 can be reclassified in the CAZy (Carbohydrate‐Active enZYmes) database, which describes the families of structurally related catalytic and carbohydrate‐binding modules (or functional domains) of enzymes that degrade, modify or create glycosidic bonds (www.cazy.org).

## Experimental methods

### Expression and purification

A synthetic construct coding for polyhistidine‐tagged Cwp19 without the N‐terminal signal peptide and C‐terminal cell wall‐binding domains (residues 27‐401) cloned into pET28a as previously described 45 was expressed in *E. coli*. 10 mL LB overnight cultures supplemented with 50 μg·mL^−1^ kanamycin were used to inoculate 500 mL LB cultures supplemented with kanamycin which were grown with shaking at 200 r.p.m. and 37 °C to an OD_600_ of 0.6–0.8. Overnight expression at 16 °C was induced by addition of 1 mm IPTG before cultures were harvested by centrifugation at 8000 *g* and flash freezing in liquid nitrogen for storage at −80 °C.

Cell pellets were resuspended in lysis buffer (25 mm Tris, 200 mm NaCl, 40 mm imidazole, pH 8.0) and lysed at 20 KPSI in a French press. Lysate was cleared by centrifugation at 64 000 *g* and the supernatant loaded on to a nickel affinity chromatography column pre‐equilibrated with lysis buffer. The column was washed with lysis buffer before Cwp19‐fr was eluted with a single step increase in imidazole concentration to 200 mm. The imidazole was removed using a desalting column.

Selenomethionyl‐protein was produced by inhibiting methionine production as previously described 41. Buffers used for IMAC had 2 mm DTT added, while the desalting buffer had 5 mm reduced glutathione added to prevent loss of anomalous signal through oxidation 46.

### Crystallographic studies

To avoid previously identified issues with data that may have resulted in the unsuccessful molecular replacement 45, crystallisation conditions were rescreened using an Art Robbins Phoenix nano dispenser at a range of protein concentrations. The only condition identified that produced crystals that diffracted to a usable resolution, Molecular Dimensions Heavy and Light (H&L) condition H11 (50 mm KH_2_PO_4_, 14% PEG 8000), was similar to the previously identified condition. This condition produced crystals that diffracted to 2 Å with a moderately high anisotropic delta‐B of 19.6 Å^2^. To improve the quality of diffraction, the identified condition was screened around using a Protein BioSolutions OptiMatrix Maker and supplemented with a range of other screens at a concentration of 10%.

Two additive conditions were identified that produced crystals that diffracted to a higher resolution with reduced anisotropy: Molecular Dimensions Morpheus (M1) condition F7 (120 mm monosaccharides, 100 mm HEPES/MOPS pH 7.5, 40% glycerol, 20% PEG 4000) and Morpheus II (M2) condition F7 (100 mm Monosaccharides II, 100 mm BES/TEA pH 7.5, 40% pentane‐1,5‐diol). The crystal used for the high resolution native structure was obtained in a drop containing 90% (10 mm KH_2_PO_4_, 18% PEG 8000) and 10% M1 F7 mixed 1:1 with protein at 40 mg·mL^−1^. These conditions resulted in a change of space group from the primitive monoclinic cell observed for H&L H11 to a primitive orthorhombic cell. Attempts at molecular replacement using these data still failed, so a selenomethionine derivative was expressed, purified and crystallised in similar conditions. Crystals used for Se‐SAD were obtained in drops containing 90% H&L H11 with 10% M2 F7 mixed 1:2, protein:reservoir, with protein at 53 mg·mL^−1^.

Crystals were cryo‐protected by addition of PEG 8000 to a final concentration of 35‐40%. Native data were collected from a single crystal with a high resolution sweep and a low resolution sweep on beamline I02 at Diamond Light Source, while Se‐SAD data were collected on I04 using the mini‐kappa goniometer to maximise anomalous signal 47. For the SAD data, three datasets containing 9999 images each with oscillation angles of 0.1° for a total of 2999.7° of data (175 GB) were collected from two crystals. Data were indexed and integrated with XDS 48 using Xia2 pipeline 3dii 49, 50. The three integrated datasets were scaled together with XSCALE 48, before merging with AIMLESS 51. A high resolution cut‐off was selected based upon the resolution at which the anomalous signal became unusably weak. The merged data were fed into the CRANK2 pipeline 52, 53 using SFtools, SHELXC and D 54, REFMAC5 55, MAPRO, Solomon 56, Multicomb, Parrot 57 and Buccaneer 58. The number of trials or cycles for several steps was significantly increased over the default, which lead to a solution when lower numbers had been unsuccessful. Model building was completed and the structure was refined with COOT 59 and REFMAC5.

The high resolution data, in which reflections were observed up to 0.95 Å, were indexed and integrated with DIALS 60, the number of observed reflections in the dataset mandated that this be done on a computer cluster, particularly dials.refine, which required more than 128 GB RAM. The data were scaled with AIMLESS, with a high resolution cut‐off determined based on an anisotropic correlation coefficient of 0.3. Refinement was attempted at higher resolution, but this resulted in significantly higher R‐factors and noisy maps. The Se‐SAD structure was used as a model for molecular replacement with PHASER 61, the output of which was again refined using COOT and REFMAC5. Geometric restraints were relaxed somewhat relative to those recommended by Engh and Huber 62 based on recommendations by Jaskolski *et al*. 63. Phenix 64 was used to refine occupancies. The structures were validated with MolProbity 65.

### Peptidoglycan hydrolase assay

20 mg of Lyophilised *M. luteus* cells and a protease inhibitor tablet were resuspended in 40 mL of 40 mm citrate, 40 mm K_2_HPO_4_ with the pH adjusted to a range of values between 4.0 and 6.6 with KOH. Volumes of approximately 600 μL were diluted by addition of approximately 1.9 mL of buffer to a volume of 2.5 mL and a target OD_450_ of 0.6–0.65 (measured at 0.621 ± 0.032 (SD)). Samples were stirred throughout the reaction, heated to 37 °C in a quartz cuvette and covered with parafilm to reduce evaporation. The parafilm was pierced and 100 μL of Cwp19‐fr was added to a final concentration of 200 μg·mL^−1^. The OD_450_ was measured approximately every 2 s over the space of 2 h. The assay was performed three times at each pH along with a control without addition of Cwp19‐fr. The rate of reaction was assessed by calculating the change in OD_450_ over the first 3 min and over 2 h as a proportion of the starting OD_450_ minus the change in OD_450_ of the control. Each calculation used the average of five measurements to reduce noise. A positive control was also performed with lysozyme at pH 6.2 66.

### Benedict's assay

Benedict's reagent was added to a range of concentrations of glucose and maltose to confirm that a difference could be seen between a monosaccharide and a disaccharide and to determine a sensible concentration for the main assay. Solutions of 11 carbohydrates ranging from disaccharides to polysaccharides were produced at final concentrations of 0.25% for reducing sugars and agarose and 0.5% for nonreducing sugars. A volume of 500 μL of each solution was incubated for 4 h at 37 °C with Cwp19‐fr at 200 μg·mL^−1^ and without Cwp19‐fr. After incubation, 500 μL of Benedict's reagent was added and samples were incubated at 95 °C for 10 min. The absorbance of each sample was measured at 320 nm to determine the extent of copper reduction. Six replicates were measured for each carbohydrate with and without Cwp19‐fr. The breakdown of starch by amylase was used as a positive control with three replicates.

### Substrate docking

SwissDock 24 was used to model maltose, lactose, cellobiose and melibiose into the high resolution structure of Cwp19‐fr. The program was run with the most through settings, allowing flexibility in side chains up to 5 Å from the ligand.

## Conflict of interest

The authors declare that they have no conflicts of interest with the contents of this article.

## Author contributions

WJB performed protein expression, purification, all structural biology and carbohydrate‐binding experiments, analysed the structures and wrote the manuscript. JMK, AKR and CCS analysed the data and edited the manuscript. KRA conceived and supervised the study, analysed the data and edited the manuscript. All authors reviewed the manuscript.
